# The Energy of Muscle Contraction. II. Transverse Compression and Work

**DOI:** 10.3389/fphys.2020.538522

**Published:** 2020-11-12

**Authors:** David S. Ryan, Sebastián Domínguez, Stephanie A. Ross, Nilima Nigam, James M. Wakeling

**Affiliations:** ^1^Department of Biomedical Physiology and Kinesiology, Simon Fraser University, Burnaby, BC, Canada; ^2^Department of Mathematics, Simon Fraser University, Burnaby, BC, Canada

**Keywords:** muscle, energy, finite element model, compression, transverse, tissue, deformation, 3D

## Abstract

In this study we examined how the strain energies within a muscle are related to changes in longitudinal force when the muscle is exposed to an external transverse load. We implemented a three-dimensional (3D) finite element model of contracting muscle using the principle of minimum total energy and allowing the redistribution of energy through different strain energy-densities. This allowed us to determine the importance of the strain energy-densities to the transverse forces developed by the muscle. We ran a series of *in silica* experiments on muscle blocks varying in initial pennation angle, muscle length, and external transverse load. As muscle contracts it maintains a near constant volume. As such, any changes in muscle length are balanced by deformations in the transverse directions such as muscle thickness or muscle width. Muscle develops transverse forces as it expands. In many situations external forces act to counteract these transverse forces and the muscle responds to external transverse loads while both passive and active. The muscle blocks used in our simulations decreased in thickness and pennation angle when passively compressed and pushed back on the load when they were activated. Activation of the compressed muscle blocks led either to an increase or decrease in muscle thickness depending on whether the initial pennation angle was less than or greater than 15°, respectively. Furthermore, the strain energy increased and redistributed across the different strain-energy potentials during contraction. The volumetric strain energy-density varied with muscle length and pennation angle and was reduced with greater transverse load for most initial muscle lengths and pennation angles. External transverse load reduced the longitudinal muscle force for initial pennation angles of β_0_ = 0°. Whereas for pennate muscle (β_0_ > 0°) longitudinal force changed (increase or decrease) depending on the muscle length, pennation angle and the direction of the external load relative to the muscle fibres. For muscle blocks with initial pennation angles β_0_ ≤ 20° the reduction in longitudinal muscle force coincided with a reduction in volumetric strain energy-density.

## Introduction

Muscles can change in length and develop longitudinal force when they contract, and these result in external work done by the muscle. Muscles additionally expand and develop forces in transverse directions, resulting from internal work done within the muscle. However, the transverse action of muscle is rarely studied. In this paper we show how longitudinal and transverse forces and deformations of the muscle are coupled via the internal energy of the muscle, and in particular through the redistribution of energy across different forms of strain-energy potentials.

Shape changes and muscle forces occur in all three dimensions when muscles contract. As a muscle shortens, it must increase in girth or cross-sectional area in order to maintain its volume (Zuurbier and Huijing, 1993; Böl et al., 2013; Randhawa and Wakeling, 2015). Transverse expansions of contracting muscle have been reported in both animal (Brainerd and Azizi, 2005; Azizi et al., 2008) and human studies (Maganaris et al., 1998; Randhawa et al., 2013; Dick and Wakeling, 2017), and transverse forces generated internally in the muscle can “lift” weights during contraction (Siebert et al., 2014). Conversely, transverse loads that compress the muscle in its cross-section should be transferred to forces and changes in length in the longitudinal direction of the muscle.

The force that a muscle develops in its longitudinal direction is affected by the pressure and external loads applied in the transverse direction. Various researchers have used models of fibre-wound helical tubes (mimicking the endomysium and perimysium of the extracellular matrix) to explain the transfer of transverse to longitudinal forces and deformations in the muscle (Azizi et al., 2017; Sleboda and Roberts, 2017, 2019). Loading the extracellular matrix and changing its collagen fibre orientation, by increasing the volume of the semimembranosus muscle of the bullfrog using osmotic pressure, coincides with increases in the passive force in the longitudinal direction of the muscle (Sleboda and Roberts, 2017, 2019). Limiting transverse expansion of muscle by more circumferentially oriented fibres in the helix reduces the extent to which muscle can shorten, and placing a stiff tube around contracting frog plantaris muscle reduces both how much the muscle shortens and work done in the longitudinal direction (Azizi et al., 2017).

Transverse external loads act to compress passive muscle as they do mechanical work on the tissue. Compression of passive muscle has been described for isolated medial gastrocnemius in rats (Siebert et al., 2014, 2016) and for gluteus maximus (Linder-Ganz et al., 2007) and medial gastrocnemius (Ryan et al., 2019; Stutzig et al., 2019) muscles in humans. Bulging muscles oppose the transverse load when they activate and work is generated from forces that develop in the transverse direction. The work generated from these transverse forces can be thought of as “lifting work,” if it is working against gravity (see the “Materials and Methods” for the formal definition). The muscle volume-specific energy involved in this “lifting work” from the medial gastrocnemius has been approximately 1.1−1.2 × 10^3^ J m^–3^ (Siebert et al., 2014) in the rat, and 1.1 × 10^3^ J m^–3^ in humans (Stutzig et al., 2019): it should be noted that in these experiments the plungers that applied the transverse load covered only about 20% of the surface area of the muscle, and that this “lifting work” is about 2 orders of magnitude less than the work that could be done by the longitudinal muscle force (Weis-Fogh and Alexander, 1977). The force in the longitudinal direction during muscle contraction is reduced when the muscle does work to resist the transverse loads (Siebert et al., 2014, 2018; Ryan et al., 2019; Stutzig et al., 2019), and the extent of this force reduction depended on the magnitude of the transverse force rather than the pressure that was applied externally to the muscle (Siebert et al., 2016). Siebert et al. (2012) explained transverse muscle forces and bulging from previous data using a hydraulically driven model that transfers load between the transverse to longitudinal directions. They used an ellipsoidal geometry with constraints that governed anisotropy in the deformations: their model indicated that anisotropy in the connective tissue was important for the transfer of loads between the transverse and longitudinal directions.

Muscles are additionally packaged together in anatomical compartments, and they squeeze on each other as they bulge during contraction. This caused a decrease in the force from the combined quadriceps in the rabbit when compared to the sum of the individual muscle forces if they were stimulated separately (de Brito Fontana et al., 2018, 2020), although the reasons for this were not clear. Not all muscles increase in thickness during fixed-end contractions, and thus we should not expect that every muscle will squeeze into neighbouring muscles when they activate. Muscles with lower pennation angles (<15°) tend to bulge, whereas more pennate muscle may thin as they activate (Randhawa et al., 2013; Wakeling et al., 2020).

The changes in a muscle’s longitudinal force with external loads are length-dependent. For example, the force in bullfrog semimembranosus was decreased at short lengths and increased at long lengths, when compared to the resting length, when a pressure cuff was applied around the muscle to apply transverse force (Sleboda and Roberts, 2019). Sleboda and Roberts (2019) explained these findings using a helically wound model in which the muscle acts to return to a length at which the angle of the helical fibres returns to their ideal pitch of 55° (Wainwright et al., 1976), and this pitch was assumed to occur at the muscle’s resting length. In contrast, greater reductions in muscle force have been detected in human plantarflexor muscles when they are compressed while at longer lengths (knee extended; Siebert et al., 2018) than at a shorter length (knee flexed: Ryan and Siebert observations).

When muscles contract, they increase in their pennation angle both for shortening (Kawakami et al., 1998; Héroux et al., 2016) and for fixed-end contractions (Wakeling et al., 2020). Internal deformations additionally occur within muscle when it is compressed: external transverse loads cause a reduction in the mean fibre pennation angle (Wakeling et al., 2013), and a reduction to the extent the pennation angle increases when the muscle contracts (Ryan et al., 2019). Strain-energy is the energy stored by a system undergoing deformation. We previously showed that the redistribution of strain-energy potentials within contracting muscle (Wakeling et al., 2020) changes with the pennation angle, and it is likely that work done on and by the muscle generated by forces in the transverse direction would also affect the strain-energy potentials within the muscle (Wakeling et al., 2020). Thus, we would expect that the external transverse loads affect the strain-energy potentials within the muscle, that in turn could explain the changes in force in the longitudinal direction. The redistribution between the forms of energy also depends on the muscle length (Wakeling et al., 2020), and may drive an interaction between the muscle length and the force reduction that occurs with external load. However, the relation between the strain energy potentials and a possible compression-related reduction in longitudinal force has not previously been examined. It is also likely that the strain-energy potentials and the transfer of external loads on the muscles will depend on the direction of the external load relative to the fibre pennation (for example, is the muscle compressed from its top or from its side within the transverse plane).

These recent studies have shown that muscle force changes when external transverse loads are applied to the muscle, and that this effect is length dependent. Theories have proposed how transverse forces are transferred to longitudinal forces through the properties of the connective tissue in the muscle (Smith et al., 2011; Sleboda and Roberts, 2019). However, these studies have not explained how the deformation of muscle tissues due to external transverse loads is affected by the internal geometry or by the direction of the applied load relative to the muscle fibres. Our previous description of muscle (Wakeling et al., 2020), that quantifies how strain-energy potentials in the contractile elements are redistributed throughout the muscle volume and the contractile force is redirected across all three dimensions of the muscle tissue, provides a framework that is well suited to understand these mechanics of muscle compression. The purpose of this study was to identify whether the altered muscle forces that occur with compression can be explained in terms of the strain-energy potentials within the muscle, and in particular to account for the role of muscle length, pennation angle and the direction of the external load on the changes to muscle force. Here we consider purely the mechanics of muscle tissue that can be thought of as a block of muscle abstracted from a muscle belly, and that is free from influences of aponeurosis or tendon.

## Materials and Methods

In this paper we present simulations to compare the changes in the internal energy and internal pressure of muscle tissue during an external compression. We modelled the muscle as a 3D and nearly incompressible fibre-reinforced composite biomaterial. The presence of 1D fibres through the base-material, representing contractile elements, results in an overall anisotropic response of the muscle tissue. The formulation of our model used in all simulations is based on the balance of strain-energy potentials as presented in Wakeling et al., 2020, and was solved using the finite element method (FEM). The main change is that here we study the influence of external loading on muscle force output.

The internal pressure is related to the dilation in the tissue and the volumetric strain energy-density ψ_vol_ in the muscle:

(1) $$\psi_{\text{vol}}\,\left(u,p,J\right)=\frac{\kappa }{4}\,\left(J^{2}-2\,\log\left(J\right)-1\right)+p\,\left(J-I_{3}\,\left(F\right)\right),$$

and can be calculated from the first variation in the volumetric strain energy-density with respect to *J*:

$$p-\frac{\kappa }{2}\,\left(J-\frac{1}{J}\right)=0,$$

where **u** is the displacement vector, *p* the internal pressure, *J* the dilation, κ the bulk modulus of the tissue, and *I*_3_(**F**) the third invariant of the deformation tensor **F** (Wakeling et al., 2020). Note that we use strain-energy potentials to compare between blocks for the most of the discussion because the muscle blocks in this study all had the same initial volume. Muscle can show small changes in volume when it contracts (Neering et al., 1991; Smith et al., 2011; Bolsterlee et al., 2017), and so we have modelled the muscle as a nearly incompressible tissue (Wakeling et al., 2020).

### Muscle Geometries and Simulations

**FIGURE 1:**
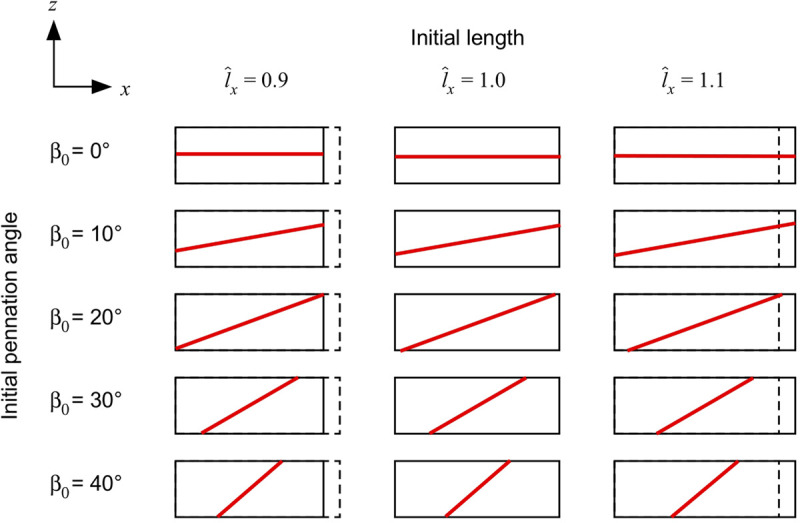
Muscle blocks used for the simulations. Muscle blocks are shown from their side view, and illustrate the different length of the blocks (the resting length of 1 is shown with the dashed line), and different fibre orientations (shown by the direction of the red line in the centre of each block).

We constructed a series of blocks of parallel-fibred and unipennate muscle with cuboid geometries (30 × 10 × 10 mm) and no aponeurosis (Figure 1). The origin of the coordinate system was centred within the blocks for their initial configuration *V*_0_. The muscle blocks had faces in the positive and negative *x*, *y*, and *z* sides. We defined the *length* of the blocks as the distance between the positive and negative *x*-faces in the *x*-direction, the *width* as the distance between the positive and negative *y*-faces in the *y*-direction, and the *thickness* as the distance between the positive and negative *z*-faces in the *z*-direction. The muscle fibres were parallel to each other and the *xz* plane in *V*_0_, but oriented at an initial pennation angle β_0_ (0–40°) away from the *x*-direction. Note that during the muscle deformation the fibres may re-orientate to have a component in the *y*-direction. During each step of the simulations, the current pennation angle is given by the angle of the fibres relative to the longitudinal *x*-axis. We set the initial length of the fibres to their optimal length (λ_iso_ = 1), and the normalised muscle length ${\hat{l}}_{x}$ to 1 for the undeformed blocks in their initial configuration *V*_0_. The fibre-reinforced composite material represents fibres with active- and passive- properties (emulating the myofilaments in the muscle) and base material (that represents additional intra- and extracellular properties). We formulated the active and passive fibre curves as trigonometric polynomial and second-order piecewise polynomial fits of experimental data (Winters et al., 2011). For the base material we used a Yeoh model (Yeoh, 1993) fit to experimental data (Mohammadkhah et al., 2016). Further details are given in our previous study (Wakeling et al., 2020). We continued to use a scaling factor *s*_base_ of 1.5 for the base-material stiffness to ensure the convergence of the algorithm for muscle geometries with the lowest pennation at maximum activation (Wakeling et al., 2020). Fixing a face in a certain direction means that the displacement **u** on that face was fixed in the direction we mention.

**FIGURE 2:**
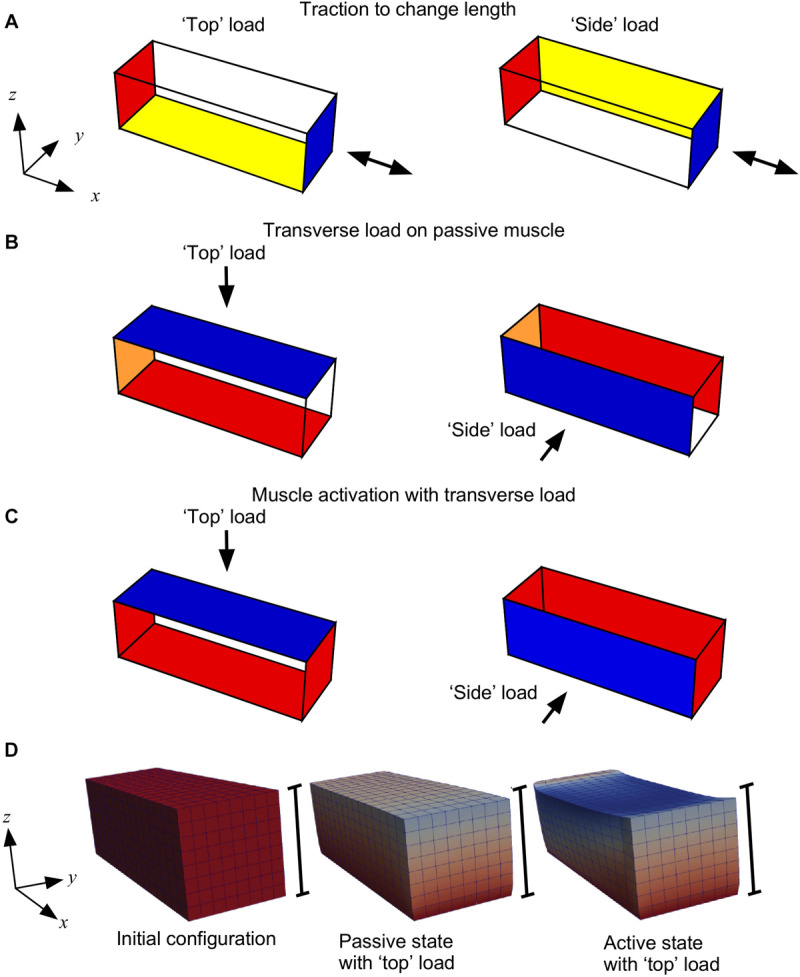
Boundary conditions for the three stages of the model. **(A)** Initially the −*x* face of the muscle block was fixed in all directions (red); for the “top” load the −*z* face was fixed in the *z* axis (yellow) and for the “side” load the +*y* face was fixed in the *y* axis (yellow); the +*x* face had traction applied (blue) to shorten or lengthen the muscle. **(B)** For compression of the muscle the −*x* face was fixed in the *x* axis (orange); for the “top” load the −*z* face was fixed in all directions (red), and a transverse load (traction) was applied to the “top” +*z* face (blue); for the “side” load the +*y* face was fixed in all directions (red), and a transverse load (traction) was applied to the “side” −*y* face (blue). **(C)** During activation the *x* faces were fixed in all directions (red), and the *y* and *z*-faces were constrained as for stage **(B)**. **(D)** Block geometries for a typical simulation. Scale bars show the height of the initial configuration, and coloration shows the deformation in the z-direction.

*In silico* simulations were conducted in a series of stages as shown in Figure 2. The different steps in our compression tests can be listed as follows:

(A) We initially fixed the −*x* face in all directions. For the “top” load we fixed the −*z* face in the *z* axis only and for the “side” load we fixed the +*y* face in the *y*-axis only. We applied a traction to the +*x* face to either stretch or shorten the passive muscle. This traction was applied in the direction normal to the +*x* face in the initial configuration *V*_0_. This stage allowed the muscle blocks to be set to different lengths. Previous experimental work studied transverse loading effects on muscle force at different muscle lengths, therefore this step in the experiments was added.
(B) Next, we changed the constraints on the −*x* face to fix it only in the *x*-direction, and we compressed the passive muscle by applying a transverse load (traction) of 0, 5, 15 or 30 kPa, consistent with previous experimental loads (Ryan et al., 2019; Stutzig et al., 2019). For the “top” load this traction was on the +*z* face and the −*z* face was fixed in all directions. For the “side” load this traction was on the −*y* face and the +*y* face was fixed in all directions. This stage is equivalent to placing an external load on the muscle, as would be the case in a physical experiment. This step was taken to determine the effects of passive muscle compression, as well as to compress the muscle before activation.
(C) Finally, we fixed both *x*-faces in all directions while maintaining the *y* and *z*-constraints and the transverse traction to compress the muscle as for step B. During this stage we ramped the activation $\hat{a}$ from 0 to 100% over a series of 10 time-steps. This stage is the main part of the study, where we could track the muscle forces that develop while the muscle is activated in its compressed state: the procedure to calculate the muscle forces has been documented in Wakeling et al. (2020). The results from the study were extracted from the simulations during this stage.

### Mechanical and Lifting Work

The work done by the muscle tissues during deformation is given in terms of the force developed by the muscle, denoted by *F*, and the displacement **u**. The total work is then defined as

$$W_{\text{int}}=\int_{V}F\cdot u\,dV,$$

where *V* is the current configuration of the muscle tissue, and the dot between the vectors denote the dot product. The external work done by the prescribed transverse loads on parts of the surface of the muscle geometries, which we denote by *S*, are computed as

$$W_{\text{ext}}=\int_{S}p_{0}\,\hat{\text{n}}\cdot u\,dS,$$

where $p_{0}\,\hat{\text{n}}$ is the transverse force and $\hat{\text{n}}$ is the normal unit vector on the surface *S*. During contraction of the muscle fibres the internal force may be greater than the force on the system from transverse external loads on the surface *S*. In such cases, one can see that the muscle surface pushes back as the transverse external load can no longer compress the tissues. The non-zero force that pushes back on the surface of the muscle, denoted by *F*_*lift*_, defines a non-zero work which is done by the tissues. We refer to this work as the “lifting work” of the muscle following Siebert et al. (2014). The “lifting work” is a form of mechanical work and is defined as

(2) $$W_{\text{lift}}=\int_{S}F_{\text{lift}}\cdot u\,dS.$$

### Post-processing and Data Analysis

The FEM model calculates tissue deformations across a set of 128,000 quadrature points within each muscle block. We defined an orientation for the fibres at each quadrature point. The pennation angle β_0_ in the undeformed and current β states were calculated as the angle between the fibre orientations and the *x*-axis: this is an angle in 3D space, similar to the 3D pennation angles defined by Rana et al. (2013). We calculated forces *F* as the magnitude of force perpendicular to a face on the muscle. The muscle force in the longitudinal direction is denoted by ${\hat{F}}_{x}$.

The internal pressure is calculated at each of the 128,000 quadrature points in the current state of the geometry through the use of Eq. 1. We compute the weighted mean pressure of the muscle block, where the weights are defined as the volume at each quadrature point divided by the total volume of the geometry in its current state. This mean internal pressure is reported in units of Pascal.

The strain-energies are initially calculated as strain energy-densities ψ, which are the strain-energy for a given volume of tissue, in units J m^–3^. We computed the total strain-energy of the tissue. The strain-energy potential *U* is the strain-energy in the tissue, in units of Joules. We calculated *U* at each given state by integrating ψ across the volume of muscle tissue at that state. We computed volumetric, muscle base-material, muscle active-fibre, and muscle passive-fibre strain-energy potentials: *U*_vol_, *U*_base_, *U*_act_, *U*_pas_, respectively (see Appendix I in Wakeling et al., 2020).

## Results

**FIGURE 3:**
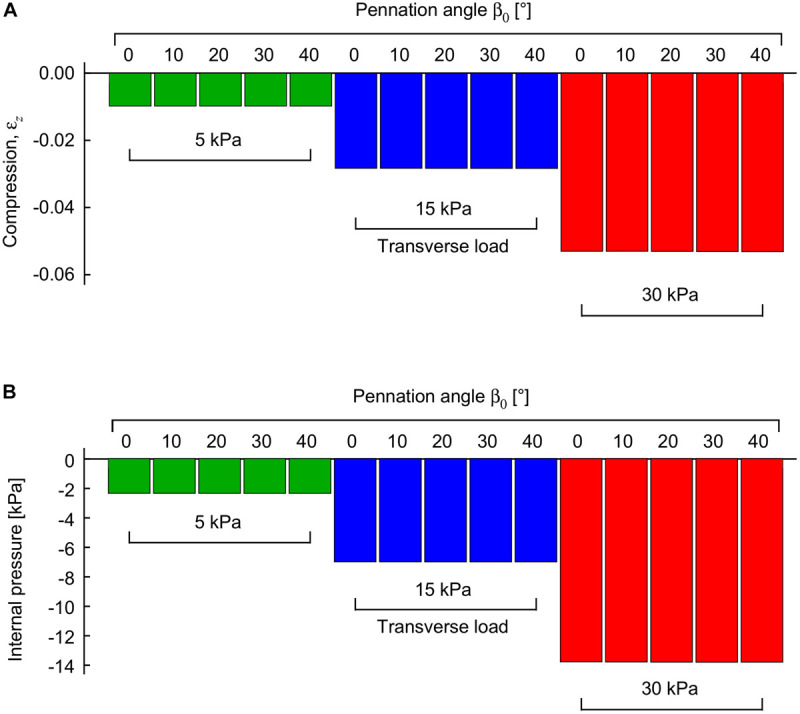
Compression and internal pressure in passive muscle blocks when transversely loaded. Transverse loads were applied in the −*z* direction to the +*z* face. Compression is shown as a strain between the *z*-faces relative to their uncompressed state **(A)**. Internal pressure is shown relative to the uncompressed state **(B)**. Transverse loads are distinguished by color (green: 5 kPa, blue: 15 kPa, red: 30 kPa). For each transverse load, different blocks were compressed with initial pennation angles β_0_ from 0 to 40°. Results are shown for blocks that were compressed at a muscle length relative to optimal length of ${\hat{l}}_{x}=1.0$.

**FIGURE 4:**
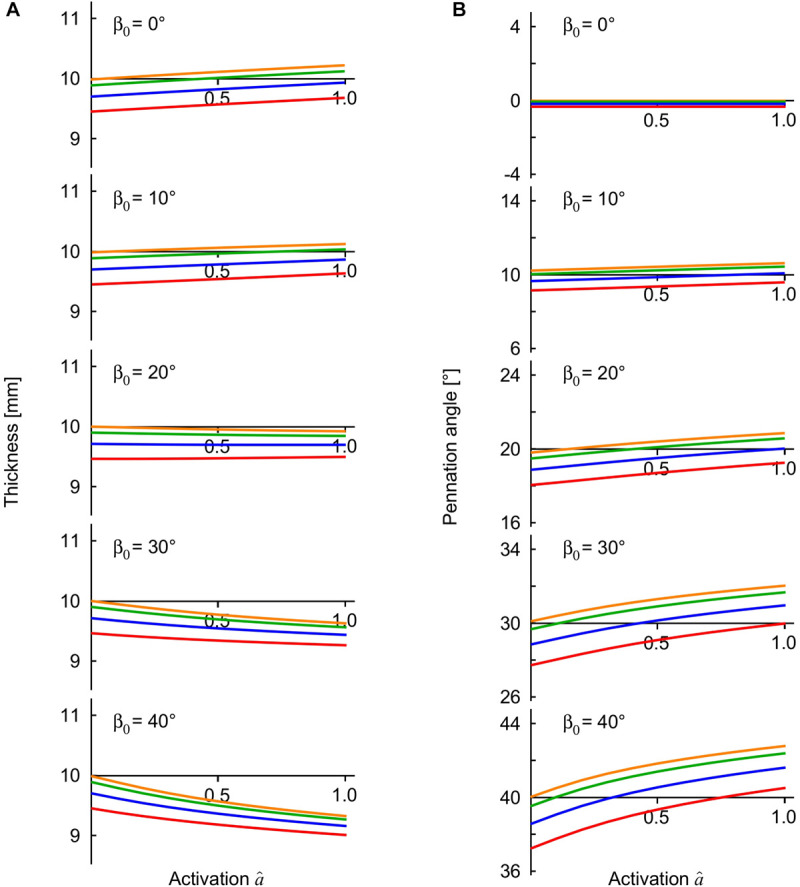
Muscle thickness and pennation angle during contractions with external loads. The transverse external loads were applied from the “top” *z* -direction and are distinguished by color (orange, 0 kPa; green: 5 kPa, blue: 15 kPa, red: 30 kPa). The thickness **(A)** is the distance between the –*z* and +*z* faces of the muscle blocks, and the pennation **(B)** is the mean pennation angle of the muscle fibres, with both these parameters being plotted against activation state for contractions against the transverse external loads. The results are shown for the muscle blocks at an initial length relative to optimal length of ${\hat{l}}_{x}=1.0$.

When the external transverse load was applied to the passive muscle blocks on the *z*-face (“top” loading), the blocks decreased in their thickness in the *z*-direction (Figure 3A), and also in their pennation angle (Figure 4). The extent of this compression increased with the external load. The increases in tissue compression with increases in external transverse load are consistent with experimental measures using compression bandages (Wakeling et al., 2013) or weighted plungers on the medial gastrocnemius (Stutzig et al., 2019), and due to sitting on the gluteus maximus in humans (Linder-Ganz et al., 2007). Note that the internal pressure in the passive muscle block decreased with increasing external transverse load (Figure 3B): whilst this may seem counter-intuitive this internal pressure is only one of the multiple factors that were balanced through the optimization of energy in these simulations (with others being the passive fibre-force, tissue volume and shape).

**FIGURE 5:**
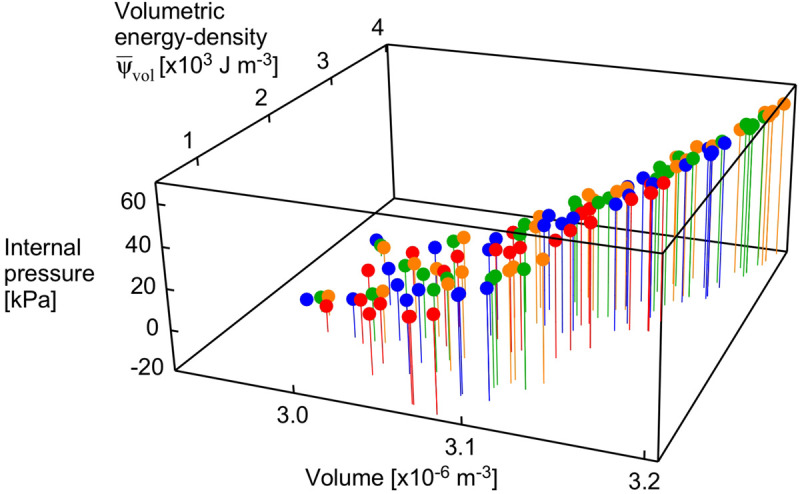
Internal pressure as a function of volume and strain energy-density. Points are shown for the maximum activation state for all muscle lengths and pennation angles. Transverse loads are distinguished by color (orange: unloaded, green: 5 kPa, blue: 15 kPa, red: 30 kPa).

The internal pressure increased as the muscle activated. The internal pressure for the maximum activation state of $\hat{a}=1$ was 31 ± 21 kPa (mean ± S.D., *N* = 120) across all geometries and transverse loads, which is within the range of intramuscular pressures measured for muscle contractions: 13–40 kPa in the frog gastrocnemius (Hill, 1948), 27 kPa for the human soleus (Aratow et al., 1993) and 30 kPa for the human tibialis anterior (Ateş et al., 2018). However, the external transverse load was not directly proportional to the increase in the internal pressure in the muscle blocks (Figures 3B, 5). Indeed, the coefficient of determination between external transverse load and the internal pressure was *r*^2^ = 0.062 for all states (*N* = 1260), and *r*^2^ = 0.046 for the fully active state (*N* = 120) in these simulations.

**FIGURE 6:**
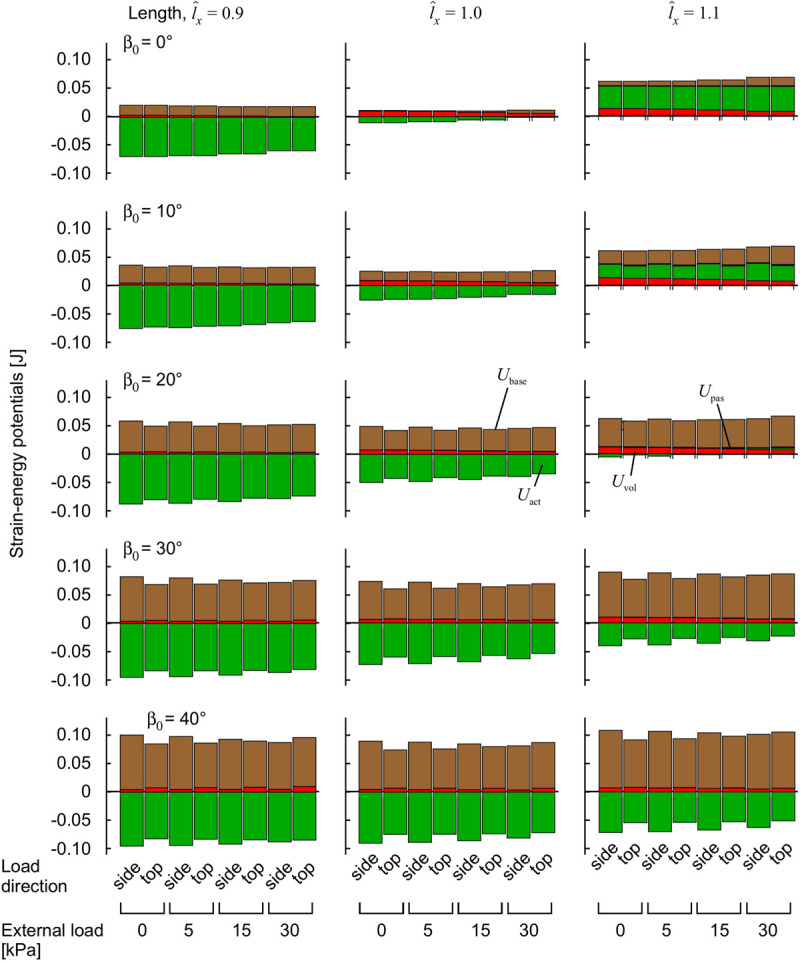
Strain-energy potentials during muscle contraction with transverse external load. The muscle blocks were initially stretched or shortened to a new length using traction on the +*x* face, then loaded in a transverse direction and activated to 100%. Strain-energy potentials are distinguished by color: base-material, brown; volumetric, red; active-fibre, green and passive-fibre, purple.

The volumetric strain energy-density varies with both muscle length and pennation angles (Figure 6) and cannot be predicted just from the activation state of the muscle (Wakeling et al., 2020). Across the range of pennation angles, activations, and muscle lengths used for the compression simulations in this study the muscle volume changed by 2% on average, and the coefficient of variation for the volume of the muscle blocks was 0.02. By contrast, the range of the volumetric strain energy-densities of the muscle blocks was much larger with a coefficient of variation of 0.97. It is this greater range of volumetric strain energy-densities that drove the changes in internal pressure of the muscle (Figure 5). Strain energy-density develops in the active fibres within the muscle (that represent the contractile elements) when the activation state increases, and is subsequently redistributed across the volumetric, base-material and passive-fibre strain energy-densities (Wakeling et al., 2020). We have also found that the volumetric strain energy-density varies with muscle length and pennation angle, and so too the internal pressure varies with length and pennation angle. This is a result of the nearly incompressibility nature of our model. As muscle tissues are allowed to change volume during contraction, the dilation *J* changes, causing the volumetric strain energy-density to change (see Eq. 1 in “Materials and Methods” section for the formal definition of this strain energy-density in terms of the dilation). Changes in both muscle length and pennation cause changes in the volume and therefore local changes in the dilation. With the definition of the internal pressure, we see that it also varies with muscle length and pennation angle. However, we noted that internal pressure does not possess a direct relation with the external transverse load: this is illustrated in Figure 5 where each given transverse load can cause a range of different internal pressures depending on the length or pennation angle of the muscle block being compressed. Note that this highlights important considerations for interpreting experimental data (Wakeling et al., 2013; Siebert et al., 2016; de Brito Fontana et al., 2018; Sleboda and Roberts, 2019) where muscle is compressed using transverse external loads, because the internal pressure in the muscle may not be directly related to the extent of the external load.

The compressed muscle blocks changed in thickness when they were activated (Figure 4A). The least pennate muscle blocks (β_0_ ≤ 10°) increased in thickness (bulged) when they were activated, and the more pennate blocks (β_0_ ≥ 20°) decreased in thickness. This difference in the direction of bulging was consistent with previous experimental (Randhawa and Wakeling, 2013, 2018; Randhawa et al., 2013; Raiteri et al., 2016; Wakeling et al., 2020) and modeling results (Wakeling et al., 2020). The parallel fibred block of muscle (β_0_ = 0°) bulged during contraction in a manner also described and discussed in Wakeling et al. (2020), however there was minimal effect of the transverse load on this bulging. By contrast, contracting against greater transverse loads increased the bulging and the changes in pennation angle of the pennate muscle blocks (Figure 4B). During contraction, all the pennate blocks “lifted” the external load when it was applied from the “top,” or in other words, they resulted in a greater distance between the *z*-faces than for the case with no transverse load. The “lifting work” done by the muscle against this external load was greatest at higher pennation angles, and reached 0.7 × 10^3^ J m^–3^ for β_0_ = 40° when measured in comparison to the unloaded state. This “lifting work” is of similar magnitude to the values recorded in experimental studies: 1.1−1.2 × 10^3^ J m^–3^ (Siebert et al., 2014) in rats, and 1.1 × 10^3^ J m^–3^ in humans (Stutzig et al., 2019), where this work is expressed as a muscle volume-specific energy density. Changes in the transverse dimensions of the muscle blocks depended on the extent of the external transverse load, the direction of that load and the pennation angle. When loaded from the “top” (*z*-) direction the contracting blocks were thinner (*z*-direction) but wider (*y*-direction) than the conditions where the external transverse load was from the “side” (*y*-) direction.

The strain-energy increased in the muscle during contraction (Figure 6). The strain-energy redistributed across different strain-energy potentials (volumetric, base-material, active-fibre and passive-fibre) in a complex manner that depended on the muscle length, activation and pennation angle, similar to our previous study (Wakeling et al., 2020), and also the magnitude and the direction of the transverse external load relative to the muscle fibres. Muscles with greater initial pennation angle β_0_ developed much larger base-material strain-energy potentials (Figure 6), due to the greater shortening of the muscle fibres, in a manner also seen in our previous study (Wakeling et al., 2020). The volumetric strain-energy potential was larger at longer muscle lengths ${\hat{l}}_{\text{x}}$ for β_0_ ≤ 20° as also shown in our previous study (Wakeling et al., 2020), but the relation was more complex at β_0_ ≥ 30° (Figure 6). The volumetric strain-energy potential was reduced with greater transverse external loads for most muscle lengths and pennation angles β_0_, apart from at β_0_ of 30–40° and ${\hat{l}}_{\text{x}}=0.9$ (Figure 6).

**FIGURE 7:**
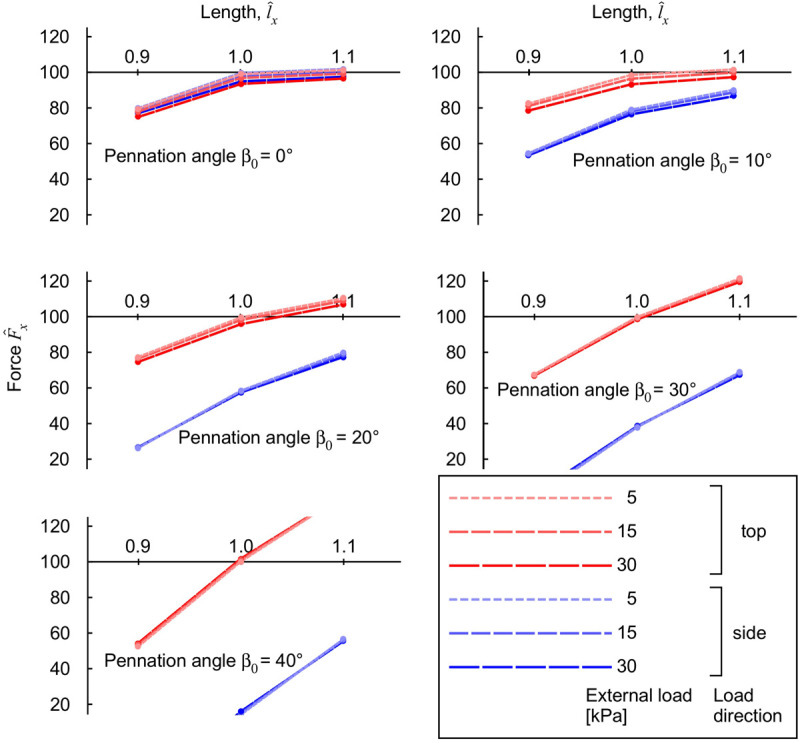
Longitudinal muscle force during contraction with transverse load. Transverse loads are distinguished by load direction (top: red and side: blue), and transverse load (color intensity and dashing). For each transverse load, different blocks were compressed with initial pennation angles β_0_ from 0 to 40°. The longitudinal force ${\hat{F}}_{x}$ is normalised to the maximum force achieved for the unloaded muscle at normalised length ${\hat{l}}_{x}=1.0$, for each initial pennation β_0_.

The longitudinal muscle force ${\hat{F}}_{\text{x}}$ varied with muscle length, pennation angle and the magnitude and direction of the transverse external load (Figure 7). For comparative purposes, longitudinal force ${\hat{F}}_{\text{x}}$ is expressed relative to the maximum uncompressed longitudinal force for that muscle at its resting length ${\hat{l}}_{\text{x}}=1.0$ and pennation angle β_0_. We also compared here the results for transverse loading from the “top” (*z*-) and the “side” (*y*-) direction (Figure 2). The longitudinal force ${\hat{F}}_{\text{x}}$ was reduced with transverse load at all muscle lengths for the β_0_ = 0° block, with greater reductions occurring at higher transverse loads. For blocks with β_0_ ≥ 20° there was a length-dependency to the effect of transverse load, with the force ${\hat{F}}_{\text{x}}$ being reduced at short lengths, and enhanced at long lengths for the “top” loaded condition. Applying the external load from the “side” resulted in reductions in longitudinal force ${\hat{F}}_{\text{x}}$ for all muscle lengths and pennation angles, with these reductions getting more pronounced as the initial pennation angle increased. Thus, the results show that a muscle’s longitudinal force changes with transverse external load in a complex manner that depends on the length, pennation angle and direction of the transverse load. This effect being due to the way in which the strain-energy potentials are redistributed across the muscle during these transversely loaded contractions (Figure 6).

**FIGURE 8:**
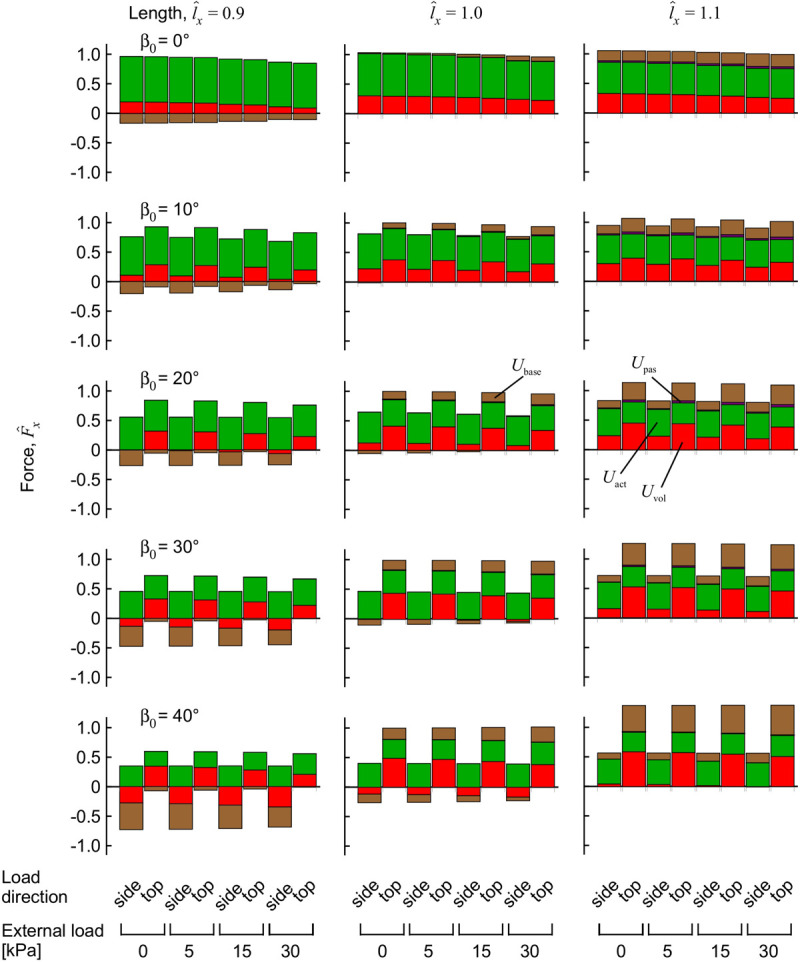
Components of force from the strain-energy potentials during muscle contraction with transverse external load. Longitudinal force for the muscle blocks, measured on the *x* face. The muscle blocks were initially stretched or shortened to a new length using traction on the +*x* face, then loaded in a transverse direction and activated to 100%. The force ${\hat{F}}_{\text{x}}$ is normalised to the maximum force achieved for the unloaded muscle at length ${\hat{l}}_{x}=1.0$, for each initial pennation β_0_.

The contributions of the different strain-energy potentials to the longitudinal force ${\hat{F}}_{\text{x}}$ are shown in Figure 8. For muscle with low to moderate initial pennation β_0_ ≤ 20°, the largest reduction in force that occurred with external transverse load was from a reduced contribution from the volumetric strain-energy potential. At the highest pennation angles the strain energy potentials were similar between the “top” loaded and “side” loaded conditions (Figure 6); however, the contribution of the volumetric and base material strain-energy potentials to the longitudinal force was markedly different (Figure 8). The pronounced difference in the longitudinal force between the “top” loaded and “side” loaded conditions stemmed from the large and positive volumetric and base material strain-energy potentials for the “top” load at the longer lengths, and the large but negative volumetric and base material strain-energy potentials for the “side” load at the shorter length.

## Discussion

The longitudinal force developed by a muscle depends on a range of factors. The myofilaments of the contractile elements within the muscle fibres develop forces from elastic proteins such as titin when they are stretched, and additionally from cross-bridges between the actin and myosin filaments that form when the muscle is active (Rode et al., 2009; Herzog et al., 2012; Nishikawa et al., 2012). The myofilaments are typically considered as one-dimensional actuators (Zajac, 1989), with their forces being directed along the length of the muscle fibre; however, more recent models have considered radial forces that develop from both cross-bridges (Williams et al., 2010) and from titin (Nishikawa et al., 2012). The myofilaments are embedded within the sarcoplasm of the muscle fibres, and the muscle fibres are part of the muscle belly held connected through the extensive extracellular matrix. These cellular and extracellular materials confer volume and shape regulating properties on the muscle tissue, they have been represented as homogeneous (Rahemi et al., 2015; Spyrou et al., 2017) or fibre-reinforced (Sharafi and Blemker, 2010; Gindre et al., 2013; Azizi et al., 2017; Sleboda and Roberts, 2019) materials and they affect the 3D properties of the muscle tissue deformation. In this study we include the effect of external transverse loads on the muscle: these loads apply forces on the cellular and extracellular components through their volumetric and base material properties, which in turn transfer the forces to the contractile elements. While muscle experiments traditionally considered muscles contracting in isolation, more recently studies have examined the influence of surrounding tissues (de Brito Fontana et al., 2018, 2020) and transverse external loads (Siebert et al., 2014, 2018; Ryan et al., 2019; Sleboda and Roberts, 2019; Stutzig et al., 2019) on the longitudinal muscle forces. Here we show how the mechanics of these external transverse loads affects the energy distribution and longitudinal forces developed by the muscle.

In this study we used the FEM to evaluate a 3D model of skeletal muscle, based on the principles of continuum mechanics, to probe the relation between external transverse load on the muscle and the force that it can develop in its longitudinal direction as well as the work done by the muscle. The FEM model contained a series of constitutive relations that are based on phenomenological descriptions of contractile elements and tissue properties (see details of the model in Wakeling et al., 2020): none of these relations were specifically derived from or optimised to the transverse response of muscle contractions, in contrast to previous models (Siebert et al., 2012, 2014, 2018; Randhawa and Wakeling, 2015). Nonetheless, the model predicted many of the general features of the compression response that have been previously reported, and so these general features emerge from the physical principles that govern 3D deformations in muscle tissue. We chose the main direction of the transverse load to be from the “top” which is an external load acting parallel to the plane of the muscle fibres and is the direction that was tested in previous uniaxial loading in both animal (Siebert et al., 2014, 2016) and human experiments (Ryan et al., 2019; Stutzig et al., 2019). With this compression from the top, our model predicted that passive muscle tissue would decrease in thickness (Figure 3), and the fibres would decrease in pennation angle, supporting experimental results (Ryan et al., 2019). When the compressed muscle was activated, the model predicted that it would increase in thickness to “lift” the external load.

The model in this study highlights the dependency of muscle length and pennation angle on the changes in longitudinal muscle force with applied transverse loads. We found that muscle blocks with shorter muscle lengths and low pennation angles showed a force reduction with transverse load (Figure 7), however, longer muscles and higher pennation angles resulted in increased longitudinal force. The length dependency derives from both the fibre and the base properties of the muscle model. The fibres are encoded as contractile elements that have length-dependent force properties for both the active-fibre and passive-fibre components, and the base properties are governed by both the volumetric and base-material relations (Rahemi et al., 2014; Wakeling et al., 2020). The combination of the volumetric and base-material properties results in a tissue that tends to return to its initial state (volume and shape) after it has been deformed, and this is a similar property to the helical-wound representation of connective tissue modelled by Sleboda and Roberts (2019). Indeed, our general finding of force increase at longer lengths and force reduction at shorter lengths, due to applied transverse load, matches these previous findings (Sleboda and Roberts, 2019), although our models shows that an isotropic base-material is sufficient to explain these properties. It should be noted that the initial undeformed state that these models return to is a discretionary choice between studies, and so it should not be expected that the exact same length-dependency of the force reduction due to external transverse load would occur across the different models. Indeed, this is the case where the helical model predicts force increase at longer lengths (Sleboda and Roberts, 2019), whereas our model predicts these increases at the largest pennation angles (Figure 7).

The simulations presented in this paper show that the pennation angle of the muscle had a pronounced effect on the muscle response to compression in terms of tissue deformation (Figure 4), strain-energy potentials (Figure 6), and the changes in muscle force (Figure 7). When pennate muscle contracts, the fibres rotate to greater pennation angles as they shorten (Fukunaga et al., 1997; Maganaris et al., 1998). Muscle fibres act to draw the aponeuroses together (or for these simulations, the *z*-faces) as they shorten, which tends to decrease the muscle thickness. However, the fibres increase in girth during shortening in order to maintain their volume (Rahemi et al., 2014, 2015). The increase in girth may be in either the width or thickness direction, and indeed the relative deformations may vary between muscles (Randhawa and Wakeling, 2015, 2018) due to stress asymmetries through the muscle (Wakeling et al., 2020). However, a general effect is for the muscle to increase in pennation angle to allow their fibres to fit within the enclosed volume of the muscle tissue (Zuurbier and Huijing, 1993; Fukunaga et al., 1997). This increase in pennation angle tends to increase the muscle thickness (Randhawa and Wakeling, 2018), which in turn resists muscle compression acting from the “top” direction and contributes to the “lifting work” of the muscle. We additionally show how the strain-energy potentials redistribute within the muscle in a pennation-dependent manner (Figure 6; Wakeling et al., 2020). Thus, the response to the compression and the work done by the muscle will also be pennation dependent, due to the altered balance of strain-energy potentials within the muscle. We show here that the changes in longitudinal force that occur with transverse loading of the muscle seem particularly dependent on the volumetric and base-material strain-energy potentials (Figure 8), that in turn vary with pennation angle and the direction of the external load relative to the fibres (“side” or “top”: Figure 6).

Strain-energy potentials develop during contraction and are distributed through the muscle (Wakeling et al., 2020). When the muscle contracts it increases in its free energy, with this energy being derived from the hydrolysis of ATP to ADP within the muscle fibres (Woledge et al., 1985; Aidley, 1998). The active-fibre strain-energy potentials are redistributed to passive-fibre strain-energy potentials and then to the base material strain-energy potential that develops in the bulk muscle tissue within the muscle fibres (excluding the myofilament fraction), connective tissue surrounding the muscle fibres such as the extracellular matrix, and in sheets of connective tissue that form the aponeuroses and internal and external tendons. Energy is also used to change the muscle volume. Whilst muscle is often assumed to be incompressible, small changes in volume can occur in fibres (Neering et al., 1991), bundles of fibres called fascicles (Smith et al., 2011), and in whole muscle (Bolsterlee et al., 2017). The volumetric strain-energy potential, which accounts for an energetic penalty to any changes in volume that occur, builds up as the muscle is activated and shows slight increases in volume (Wakeling et al., 2020). The transverse external loads in this study act to compress the volume of the muscle (Figure 3A). These changes in volume relate to changes to the volumetric strain-energy potential as the muscle is compressed. The volumetric strain-energy potential associates with the contractile force *F*_x_ in the longitudinal direction (for a description of the relation between strain-energy potentials and force, see Wakeling et al., 2020) for all except the highest pennation angles and shortest muscle lengths. Thus, the compression-induced reductions in volumetric strain-energy potential result in the reductions to force in the longitudinal direction during the muscle contractions (Figure 8).

The volumetric strain-energy potential is arguably the least-well characterised component of the internal energy in the muscle in our simulations. The extent of the change in volume and the volumetric strain-energy potential is related to the choice of the bulk modulus κ of the tissue. A constitutive equation to calculate the volumetric strain-energy potential has not been defined for muscle tissue, and so we used a general form (Eq. 1) that is used for compressible neo-Hookean materials (see, e.g., Pelteret and McBride, 2012). Here we used a value of κ = 10^6^ Pa that was consistent with previous studies (Rahemi et al., 2014, 2015, Wakeling et al., 2020). We previously showed that this κ resulted in volume changes of 2–4% during contraction of fully active parallel muscle fibres, and in this study resulted in a mean volume change of 2%. Nonetheless, a previous study showed that κ can be varied across a wide range of magnitudes and still result in similar predictions of tissue deformation (Gardiner and Weiss, 2001). Given the apparent importance of the volumetric strain-energy potential to the modulation of contractile force in response to muscle compression, establishing muscle-specific constitutive equations for the volumetric strain energy-density and values for the bulk modulus will be an important area of future investigation.

## Conclusion

(1) We used a 3D model of muscle, represented as a fibre-reinforced composite biomaterial, to quantify the strain-energy potentials within the muscle whilst it contracted under the influence of an external transverse load. The external transverse load affects the balance of the energy between the volumetric, base material and fibre strain-energy potentials.
(2) When a block of muscle fibres with a zero pennation angle contracts, while an external transverse load is applied, its longitudinal force decreases when compared to conditions with no transverse load. The decrease in longitudinal force is dependent on the magnitude of the transverse load and the muscle fibre length. The transverse load resists the natural tendency of the muscle to bulge (expand in the transverse plane), and the ensuing decrease in its volumetric strain-energy potential contributes to the reduced longitudinal force.
(3) When pennate muscle contracts with external transverse loads then the length-dependency on the longitudinal force becomes more pronounced than for the parallel-fibred case (zero pennation angle). When the external load is from the “top” (which is a transverse direction parallel to the initial plane of the muscle fibres) then the longitudinal force increases at muscle lengths longer than the resting length, and decreases for shorter lengths.
(4) When pennate muscle contracts with a transverse external load from the “side” direction then the longitudinal force decreases, when compared to conditions with no transverse load, for all muscle lengths and pennation angles tested. The differences in the response to “top” and “side” loading occurred despite similar strain-energy potentials for these conditions but because they had different effects on the longitudinal force. These differences in the response to “top” and “side” loading were likely caused by the differences in the deformations in thickness and width that occurred with these loading directions.

## Nomenclature

**Activation** specifically refers to the active *state* of the contractile elements (muscle fibres), and is used to scale the active force that they can develop.

**Muscle contraction** is the *process* of muscle developing forces when its activation level is greater than zero. In muscle physiology, contraction does not necessarily mean shortening because tension can be developed without a change in length.

**Fixed-end** is used to refer to all the muscle block simulations where the blocks had their +*x* and −*x* faces fixed, and the distance between these faces did not change when the activation increased. It is recognised that during these contractions the local fibre stretch varied through the block, and was not always equal to one, and therefore these fixed-end contractions were not isometric at the level of the contractile fibres at each quadrature point.

The **longitudinal direction** is the major *x*-axis of each muscle block. This can be considered the direction that would be between the proximal and distal tendons in a fusiform muscle, and so it is in the commonly phrased “line of action.” We do not use “line-of-action” (except when referencing sarcomere properties), because forces and deformations occur in 3D in this study and so there is no unique line-of-action.

**Transverse direction** is used to describe directions in the *y-z* plane, and thus is perpendicular to the longitudinal direction of the muscle block. This is sometimes called the radial direction in other studies.

**Muscle bulging** is a term used to describe the muscle increasing in its girth, which is in the transverse *yz*-plane. Bulging can occur with expansions in either the *y*-direction (**width**) or *z*-direction (**thickness**).

**Force** and **load**. In this paper we use the term force for the forces developed in the longitudinal *x*-direction. The term load is used for the external transverse forces that are applied to the muscle in the *y-z* plane.

**Compression** is used to describe the process of applying the transverse load to the muscle.

**Top and side**. In the undeformed state the muscle fibres are aligned in the *xz*-plane. Top loading refers to conditions when the compression is applied in the -*z*-direction, and side loading refers to conditions when the compression is applied in the +*y*-direction.

**Lifting force and work**. Assume a weight is used to apply a transverse load on the muscle. Muscle fibres are capable of producing large non-zero internal forces when they contract that can lift the weight. This non-zero force is referred to as “lifting force.” The “lifting work” is defined as the work done by this lifting force (see Eq. 2).

**Internal and hydrostatic pressure**. Internal pressure is defined as the change in the volumetric strain energy-density with respect to the dilation *J*. This has a different definition from the hydrostatic pressure on an object, that is commonly measured experimentally, which is defined as the as total gravitational force per unit of area caused by the amount of fluid mass on such object.

**Fixed face**. A face being fixed in some direction refers to the displacement **u** of the tissues on that face being fixed in that particular direction.

## Data Availability Statement

The datasets generated for this study are available on request to the corresponding authors.

## Author Contributions

DR, NN, and JW contributed to the study design. DR, SD, SR, NN, and JW contributed to the model development. DR ran all the simulations for the manuscript and data analysis. DR and JW contributed to the first draft of the manuscript. DR, SD, SR, NN, and JW contributed to final manuscript preparation.

## Conflict of Interest

The authors declare that the research was conducted in the absence of any commercial or financial relationships that could be construed as a potential conflict of interest.
